# Proteomics analysis reveals protein expression differences for hypopharyngeal gland activity in the honeybee, *Apis mellifera carnica* Pollmann

**DOI:** 10.1186/1471-2164-15-665

**Published:** 2014-08-08

**Authors:** Ting Ji, Zhenguo Liu, Jie Shen, Fang Shen, Qin Liang, Liming Wu, Guohong Chen, Miguel Corona

**Affiliations:** College of Animal Science and Technology, Yangzhou University, Yangzhou, Jiangsu China; College of Bee Science, Fujian Agriculture and Forestry University, Fuzhou, China; Institute of Apiculture Research, Chinese Academy of Agricultural Sciences, Beijing, China; USDA-ARS Bee Research Laboratory, Beltsville, MD USA

**Keywords:** Hypopharyngeal gland, Quantitative proteomics, iTRAQ, Secreted protein

## Abstract

**Background:**

Most of the proteins contained in royal jelly (RJ) are secreted from the hypopharyngeal glands (HG) of young bees. Although generic protein composition of RJ has been investigated, little is known about how age-dependent changes on HG secretion affect RJ composition and their biological consequences. In this study, we identified differentially expressed proteins (DEPs) during HG development by using the isobaric tag for relative and absolute quantification (iTRAQ) labeling technique. This proteomic method increases the potential for new protein discovery by improving the identification of low quantity proteins.

**Results:**

A total of 1282 proteins were identified from five age groups of worker bees, 284 of which were differentially expressed. 43 (15.1%) of the DEPs were identified for the first time. Comparison of samples at day 6, 9, 12, and 16 of development relative to day 3 led to the unambiguous identification of 112, 117, 127, and 127 DEPs, respectively. The majority of these DEPs were up-regulated in the older worker groups, indicating a substantial change in the pattern of proteins expressed after 3 days. DEPs were identified among all the age groups, suggesting that changes in protein expression during HG ontogeny are concomitant with different states of worker development. A total of 649 proteins were mapped to canonical signaling pathways found in the Kyoto Encyclopedia of Genes and Genomes (KEGG), which were preferentially associated with metabolism and biosynthesis of secondary metabolites. More than 10 key high-abundance proteins were involved in signaling pathways related to ribosome function and protein processing in the endoplasmic reticulum. The results were validated by qPCR.

**Conclusion:**

Our approach demonstrates that HG experienced important changes in protein expression during its ontogenic development, which supports the secretion of proteins involved in diverse functions in adult workers beyond its traditional role in royal jelly production.

**Electronic supplementary material:**

The online version of this article (doi:10.1186/1471-2164-15-665) contains supplementary material, which is available to authorized users.

## Background

In addition to the benefits of the highly eusocial honeybee (*Apis mellifera*) in pollination and other aspects of ecology, the honeybee has been used as a model organism in studies of development, cognition, and neuroscience. Furthermore, bees are widely recognized for their production of valuable substances, including honey, royal jelly (RJ), propolis, and other products that have the potential to be used as drugs or ointments [1].

Royal jelly is a protein-rich secretion that serves as food for larvae and adult queen honey bees. Hypopharyngeal glands (HGs) are important exocrine glands localized in the anterior part of the head of bees [2]. The HG is constituted by hundreds of acini that are attached to an axial duct that opens onto the suboral plate of the hypopharynx [3, 4]. Morphologically, the acini of the HGs change in size radically with age [5], with a peak size at approximately 6 days that decreases after day 15 during the summertime [6]. The amount of RJ secreted by the secretory cells is also positively correlated with size of the acini [5], with undeveloped or hypertrophied cells having less activity than glands of medium size. According to the age polyethism of bees, there are two described phases of the secretory cycle: production of RJ, followed by production of enzymes such as α-glucosidase, which increases with the age of the worker bee.

The main secretory product of the HG is the important functional food RJ [7] and the major ingredients of the various proteins have drawn the attention of researchers interested in disease therapy, health protection, immunity, and other areas. RJ is a yogurt-like milk substance that is produced by nurse bees (typically young workers) used to nourish workers during the first 3 days of larval development and queens throughout their entire life. Irrespective of geographical and climatic conditions, RJ typically contains multiple components, including proteins (12-15%), sugars (10-12%), lipids (3-7%), minerals, vitamins [8], salts, and amino acids [9, 10] and specific vital factors that act as biocatalysts in cell regeneration processes within the human body [11]. Lipids present in RJ are secreted from the mandibular glands, while most of the proteins contained in the RJ are secreted from the HG [12, 13]. The majority of the proteins in the RJ (82-90%) belong to the major royal jelly protein family (MRJPs 1–9) [12, 14]. MRJP1 is the most abundant of them, representing 48% of the water-soluble proteins [15]. RJ has diverse nutritional and/or pharmacological functions, such as hypotensive activity, antitumor activity, insulin-like action, and disinfectant action [16–18]. Although RJ protein composition has been described in several studies, potential developmental changes in glandular secretion had not been considered.

Recent advances in chromatography, mass spectrometry, and bioinformatics have allowed significant progress in the area of quantitative proteomics [19, 20]. Previous proteomic researches on the honeybee mainly used gel-based 1D/2D gel electrophoresis technique [6, 21] for protein identification and characterization. Though the electrophoretic approach is still widely used, it is laborious and prone to experimental errors [22]. Furthermore, the lack of accuracy of 1D/2D methods for the identification of low-abundance proteins, such as membrane proteins and other hydrophobic proteins, has partly restricted their application [23–25]. The isobaric tag for relative and absolute quantification (iTRAQ)-based quantitative proteomic strategy to assess proteome-wide expression profiling has facilitated the detection of new proteins and enhanced the sensitivity of screening the proteome [26, 27]. iTRAQ combined with multidimensional liquid chromatography (LC) and tandem mass spectrometry (MS) analysis [28] is emerging as a powerful method in the search for disease-specific targets, such as for lung adenocarcinoma [29], metastatic hepatocellular carcinoma [30], sepsis prognosis [31], and colorectal cancer [32].

Our knowledge of iTRAQ, a widely employed method in proteomic workflows, has expanded dramatically in the short time since its invention [33]. iTRAQ combined with two-dimensional LC-tandem MS (2D LC-MS/MS) is one of most powerful tools in quantitative proteomics [34, 35] and novel biomarker discovery [36]. To gain insights into the molecular mechanisms involved in HG development, we combined the iTRAQ method with MS/MS to identify age-dependent changes in HG protein expression. The identification of a significant number of differentially expressed proteins (DEPs) during HG development provides new insights into the molecular basis of HG development and RJ function.

## Methods

### Preparation of protein samples

In natural conditions, honey bee queens mate with an average of 12 drones [37]. This leads to a genetically composite colony compromised of several worker subfamilies fathered by different drones [38]. We restricted the genetic background of the workers used in this study by single-drone inseminated queens [39], which were obtained from the Apiculture Science Institute of Jilin Province, China, and raised in the apiaries of Yangzhou University in June 2012.

One thousand newly emerging bees were painted and introduced into one typical host colony. Groups of 30 marked bees were collected directly from nest combs after 3, 6, 9, 12, and 16 days and immediately preserved in liquid nitrogen. Collections were performed from June 10th to June 29th, 2013. Individual bees were dissected using a binocular microscope that was chilled by dry ice. The tissues were transferred into liquid nitrogen and stored at -80°C for further use.

### Protein isolation and labeling

The pooled (n = 30), frozen HG tissues as one biological replicate that were isolated on the same collection day were grounded in liquid nitrogen. Total protein was isolated from the freeze-dried powder by resuspension in 500 μl of dissolution buffer containing 1 mM PMSF, 2 mM EDTA, and 10 mM dithiothreitol (DTT). The resuspended powder was incubated for 30 min and then sonicated for 15 min. After centrifugation at 25,000 × *g* for 20 min at 15°C, the supernatant was collected and mixed with a solution of 10 mM DTT for 1 hr at 56°C to break proteins’ disulfide bonds. After centrifugation of the samples, a freshly prepared solution of 55 mM iodoacetamide (IAM) was added. The samples were wrapped in foil and incubated in the dark at room temperature for 45 min. Pre-chilled acetone was added to the protein samples at a 5:1 ratio (acetone/sample, v/v), which were then incubated at -20°C for 2 hr. After centrifugation at 25,000 × *g* for 20 min, the precipitated pellets were resuspended in 60 μL of a solution containing 7 M urea and 500 mM tetraethyl-ammonium bicarbonate (TEAB, pH 8.5) and then sonicated for 15 min. Supernatants were isolated after centrifugation at 25,000 × *g* for 20 min. Total protein concentration was measured using the Bradford method (Additional file 1: Figure S1 (A)), and protein integrity was assessed by 12% sodium dodecyl sulfate–polyacrylamide gel electrophoresis (SDS-PAGE) (Additional file 1: Figure S1 (B)).

An aliquot of 100 μg of each sample was mixed with trypsin at a final ratio of 1:20 (trypsin/sample) and then incubated overnight at 37°C. After the trypsin digestion was complete, the peptides were dried by a centrifugal vacuum concentrator, denatured with 2% SDS, reduced with reducing reagent, treated with IAM to block disulfide bond formation, and reconstituted with 0.5 M TEAB. Proteins were then labeled with the8-plex iTRAQ reagents according to manufacturer’s instructions (Applied Biosystems, MA, USA). Samples taken at day 3, 6, 9, 12 and 16 were labeled with iTRAQ reagents with molecular masses of 116, 117,118, 119 and 120, respectively. The labeled samples were mixed, incubated at room temperature for 2 hr, pooled, and then dried by vacuum centrifugation.

Next, the labeled samples were fractionated using a LC-20AB high-performance liquid chromatography (HPLC) system (Shimazu, Japan) using a 4.6 mm × 250 mm Ultremex strong cation exchange (SCX) column (Phenomenex Inc., USA). After reconstitution of the labeled peptide mixtures with 4 ml of buffer A (10 mM KH_2_PO_4_ in 25% ACN, pH 2.6), SCX separation was performed at a flow rate of 1 mL/min using elution buffer A for 10 min, followed by a linear gradient of 5–35% buffer B (25 mM NaH_2_PO_4_, 1 M KCl in 25% ACN, pH 2.7) for 11 min and 35–80% buffer B for 1 min. The eluted fractions were monitored by measuring the absorbance at 214 nm, desalted with a Strata X C18 column (Phenomenex), and finally vacuum-dried.

### Analysis of LC-ESI-MS/MS based on TripleTOF 5600

LC-ESI-MS/MS analysis was performed on a nanoACQUITY system (Waters) connected to a TripleTOF 5600 (ABSCIEX, Concord, ON). The final concentration of peptides in each fraction was approximately 0.17 μg/μl (Additional file 2: Table S1). A total of 2.25 μg (13 μl) of the peptide mixture was loaded onto a C18 BEH column (5 μm, 180 μm × 20 mm, Waters) and separated using solvent A (2% ACN, 0.1% FA, v/v) for 15 min at a flow rate of 2 μL/min. Peptides were eluted for 1 min with 5% solvent B (98% ACN, 0.1% FA) at 300 nl/min, followed by a 40 min gradient of 5-35% solvent B at 300 nl/min, a 5 min linear gradient to 80% solvent B, a maintenance with 80% solvent B for 5 min, and finally a return to 2% solvent B over 1 min.

The peptides were subjected to nanoelectrospray ionization, followed by MS/MS in a TripleTOF 5600 coupled inline to the HPLC system in reflection mode with specific applied parameters of electrospray voltage (2.5 kV) and nitrogen pressure (30 psi; 14.5 psi ≈ 1 bar). The analytical cycle consisted of a MS survey scan (400–2000 m/z) followed by 5 s of MS/MS scans (50–2000) of the five most abundant peaks (i.e., precursor ions), which were selected from the initial MS survey scan. Precursor ion selection was based on ion intensity (peptide signal intensity above 25 counts/s) and charge state (2^+^ to 5^+^). Once the ions were fragmented in the MS/MS scan, they were allowed one repetition before a dynamic exclusion period of 120 s. Intact peptides were detected at a resolution of no less than 30,000 FWHM with 10 msec accumulation time.

### Database search and quantification

The original MS/MS file data (*.wiff) was transferred to the *.mgf format and then searched against the honeybee database of NCBI and Uniport as well as the database created from the tanscriptomic CDS FASTA database (GEO accession number: GSE47136) by six-frame translation (34702 sequences) with Mascot software (Matrix Science, London, U.K.; version 2.3.02). “Target-decoy” search strategy was applied [40]. For protein identification and quantification, search parameters were set to a fragment mass tolerance (monoisotopic mass) of 0.1 Da and a peptide mass tolerance of 0.05 Da. Carbamidomethylation of cysteine (Cys) was considered a fixed modification, and the conversion of N-terminal glutamine (Gln) to pyroglutamic acid and the oxidation of methionine (Met) were considered variable modifications. The instrument type was set to “default”, and the enzyme specificity of trypsin was set to allow up to one missed cleavage. A mass accuracy of 2 parts per million (ppm) was used in this analysis, which is typical for the latest benchtop time-of-flight mass spectrometers.

All identified peptides were tested significance by the Mascot software under the threshold of 1% FDR. Proteins quantified with at least a 1.5-fold change were considered DEPs [31].

### Bioinformatics and annotations

To determine the biological and functional properties of the peptides and to identify candidate biomarkers, DEP sequences were retrieved from the UniProt database and mapped with Gene Ontology Terms (http://www.geneontology.org) by using a local blast against the FTP resource (http://ftp.geneontology.org/pub/go). Functional category analysis was performed with protein2go and go2protein. Clusters of Orthologous Groups of Proteins System (COG, http://www.ncbi.nlm.nih.gov/COG/) was employed for the functional annotation of genes from new genomes and for research into genome evolution. The KEGG databases (http://www.genome.jp/kegg/pathway.html) and GO enrichment analysis were also used.

### Validation by qRT-PCR

The expression levels of genes corresponding to 35 DEPs that expressed in common were examined by RT-PCR with three biological replicates. Gene-specific primers (GSPs, Additional file 3: File S1) were designed as mentioned elsewhere [41]. Transcript analysis during RJ secretion revealed mixed results with two sets of information. The reactions were performed using the ABI 7500 system with SYBR Green. The iTRAQ results were basically consistent with the RNA-seq data. *Actin* (AB023025) a housekeeping gene, was used for internal control gene as described in previous studies [42, 43]. The qRT-PCR data were expressed relative to the expression of *β-actin* using the 2^-△△Ct^ method, an independent-sample *t*-test available in SPSS software (Version 16.0, SPSS Inc.). Person correlation was used to access the relativity between transcript and protein expression values [44]. A *p*-value of 0.01 was used to determine statistical significance [45].

## Results

### Overview of study workflow

The main aim of this study was to identify DEPs during HG development in adult nurse honey bees. To accomplish this objective, we performed iTRAQ analysis using direct nanoflow LC-MS/MS [46]. Proteins were extracted from the HGs at five time points (on days 3, 6, 9, 12, and 16 after the adult worker bees emerged from the comb) that corresponded to specific stages of HG activity. iTRAQ labeling combined with LC-MS/MS and Mascot searches were used to identify proteins (Sample statistics are listed in Additional file 2: Table S1). A total of 193,671 spectra were generated from iTRAQ experiments at the five different time points. Based on the Mascot search results, 9660 spectra matched known spectra, 9177 spectra matched unique peptides, 3880 matched peptides, 3757 matched unique peptides, and 1282 matched proteins (Additional file 1: Figure S2 (A), Additional file 2: Table S1), respectively. The distribution of peptide lengths (Additional file 1: Figure S2 (B)), protein masses (Additional file 1: Figure S2 (C)), the number of peptides that defined each protein (Additional file 1: Figure S2 (D)), and the distribution of proteins sequences coverage (Additional file 1: Figure S2 (E)) shows the characteristics of each of the identified proteins (Additional file 3: File S2). More than 64% of the proteins included at least two peptides.

### Quantitative strategy for the identification of DEPs

**Figure 1:**
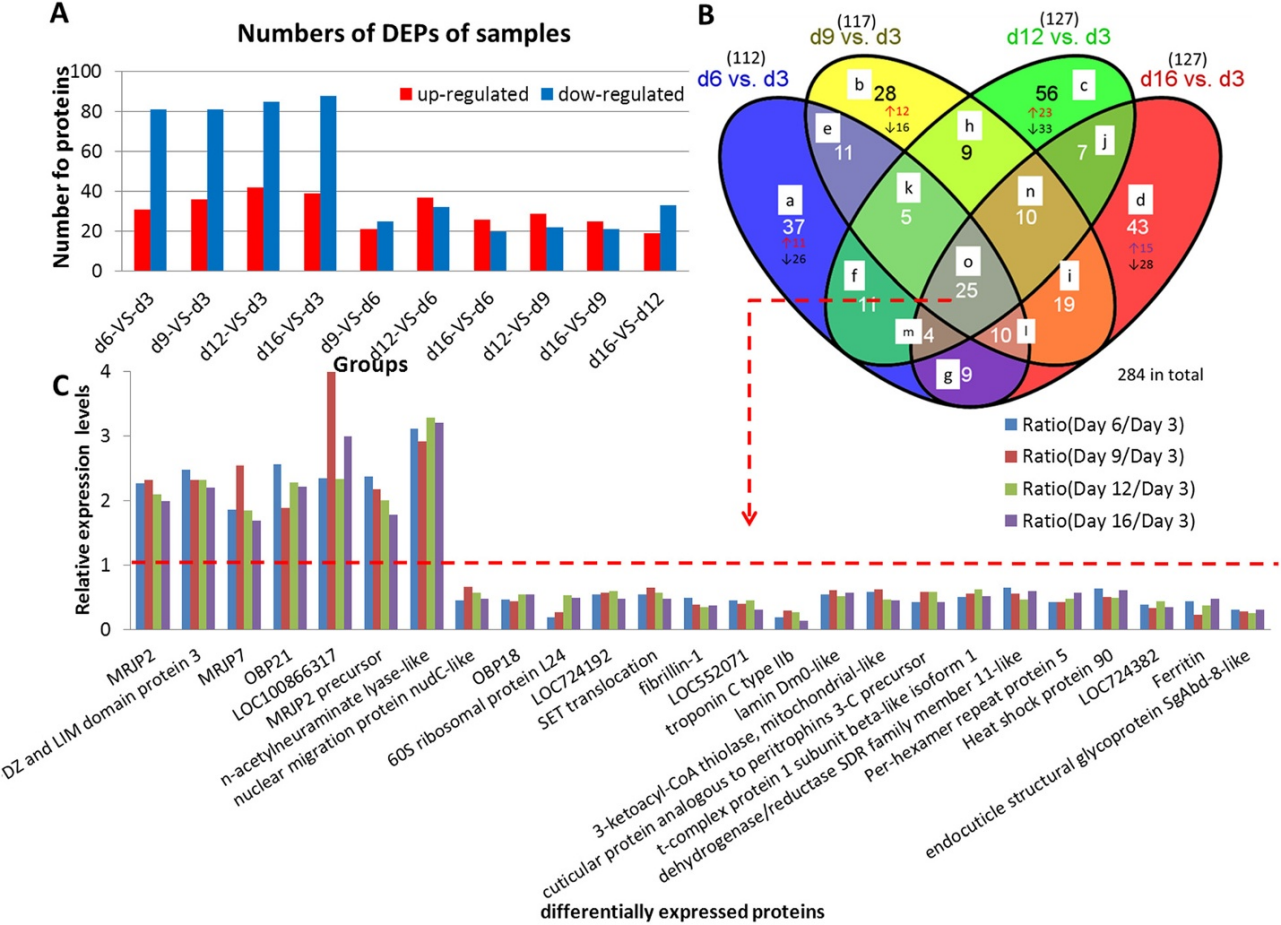
**DEPs distribution and expression levels in samples. (A)** The *x*-axis shows the pairwise comparisons of the five sample groups (days 3, 6, 9, 12, and 16), and the *y*-axis displays the number of proteins. Red and green bars indicate up-regulated and down-regulated proteins, respectively. **(B)** Venn analysis of DEPs in the five samples (Adapted from Chen *et al*. [48]). The numbers denote the amount of proteins that were expressed in each class, with arrows indicating the number of up-regulated (↑) or down-regulated (↓) proteins. Classes are labeled from **a** to **o** based on the following representations: **(a)** exclusively expressed in group d6 vs. d3; **(b)** exclusively expressed in group d9 vs. d3; **(c)** exclusively expressed in group d12 vs. d3; **(d)** exclusively expressed in group d16 vs. d3; **(e)** only expressed in group d6 vs. d3 and d9 vs. d3; **(f)** only expressed in group d6 vs. d3 and d12 vs. d3; **(g)** only expressed in group d6 vs. d3 and d16 vs. d3; **(h)** only expressed in group d9 vs. d3 and d12 vs. d3; **(i)** only expressed in group d9 vs. d3 and d16 vs. d3; **(j)** only expressed in group d12 vs. d3 and d16 vs. d3; **(k)** exclusively not expressed in group d16 vs. d3; **(l)** exclusively not expressed in group d12 vs. d3; **(m)** exclusively not expressed in group d9 vs. d3; **(n)** exclusively not expressed in group d6 vs. d3; **(o)** expressed in common. **(C)** Expression profiling of DEPs common among all the samples. The first 7 proteins were down-regulated with log2-transformed fold-change ratios that were no more than 0.5. The other proteins were up-regulated by no less than 1.5-fold. Note: It should be noted that all the redundant proteins were included in the Venn analysis.

In addition to optimization of sample preparation, a quantitative strategy to identify DEPs was employed using the mass spectrum data. DEP was decided by the Mascot software (Matrix Science, London, U.K.; version 2.3.02) with a screening criteria required a 1.5-fold change in abundance [24, 31, 47]. Based on these two criteria, 112 non-redundant DEPs (the DEPs with the same “hit number” and “score” were considered to be the same protein) were identified when comparing the day 6 group to the day 3 group. Here, sample d3 was used as the baseline for reducing the background noise corresponding to proteins unrelated to HG secretion and activity, and the other samples were compared to d3 to identify the filtered DEPs in different groups. Among these DEPs, 31 (27.7%) showed an increase and 81 (72.3%) showed a decrease in abundance. Similarly, 117, 127, and 127 DEPs were obtained when comparing the day 9, day 12, and day 16 groups to the day 3 group, respectively. These DEPs included 36 (30.8%), 42 (33.1%), and 39 (30.7%) up-regulated proteins and 81 (69.2%), 85 (66.9%), and 88 (69.3%) down-regulated proteins (Figure 1A and Additional file 3: File S3) when comparing the day 9, 12, and 16 groups to the day 3 group, respectively.

### Dynamic profiling of DEPs during HG development

Venn analysis was combined with cluster analysis of the abundance profiles for the DEP comparison groups to provide a clear visual representation of the complex cohort data. Using the Mascot search, we identified a total of 1282 proteins among all the age groups and a certain amounts of proteins with age-specific expression. Figure 1B shows the protein numbers in each class, and Additional file 2: Table S2 lists the detailed information on these proteins. Initial HG ultrastructure [3] and 2D electrophoresis analyses shows [6] that HG secretion reach its higher level between the days 6–12 and decreased it afterward.

Consistently, 28 proteins (class **b** in Figure 1B) were preferentially expressed at d9 compared to d3. Among these proteins, 12 (42.9%) were up-regulated and 16 (57.1%) were down-regulated. It should be noted that all the redundant proteins were included in the Venn analysis.

A total of 284 DEPs were identified in the five age groups, of which 43 (15.1%) proteins were novel to this study and were not annotated or predicated in the database (Additional file 2: Table S2). The bio-functions and related networks of the remaining well-described 241 (84.9%) DEPs were analyzed with bioinformatics tools.

Expression profiling of the 25 DEPs common among all the groups (class **o** in Figure 1B) is illustrated in Figure 1C and Additional file 2: Table S3 Seven of these proteins (TC12618_1, odorant-binding protein 21 precursor; TC14003_1, *N*-acetylneuraminate lyase-like (nanA); TC12484_2, fibrillin-1; TC12462_1, dehydrogenase/reductase SDR family member 11-like; TC15215_1, heat shock protein 90; TC15170_1, ferritin; and NP9546542_3, endocuticle structural glycoprotein SgAbd-2-like) (referred to as class **m**) correlated well with the HG activity and are likely involved in regulation of secretion activity.

Conversely, nine of the DEPs (TC13222_1, MRJP7; TC20339_5, uncharacterized protein LOC100866317; TC13340_1, nuclear migration protein nudC-like; TC12536_1, hypothetical protein LOC724192; TC16150_3, SET translocation; TC16851_1, troponin C type IIb; TC14021_1, 3-ketoacyl-CoA thiolase, mitochondrial-like; TC16559_2, cuticular protein analogous to peritrophins 3-C precursor; and TC12469_1, t-complex protein 1 subunit beta-like isoform 1) (referred to as class **o**) were not well correlated with the HG activity. Their predicted functions are described as follows.

### Functional and bioinformatics analyses

To identify candidate biomarkers of HG development, we analyzed the experimental results using GO annotations, COG classification, KEGG pathway, and enrichment analysis.

**Figure 2:**
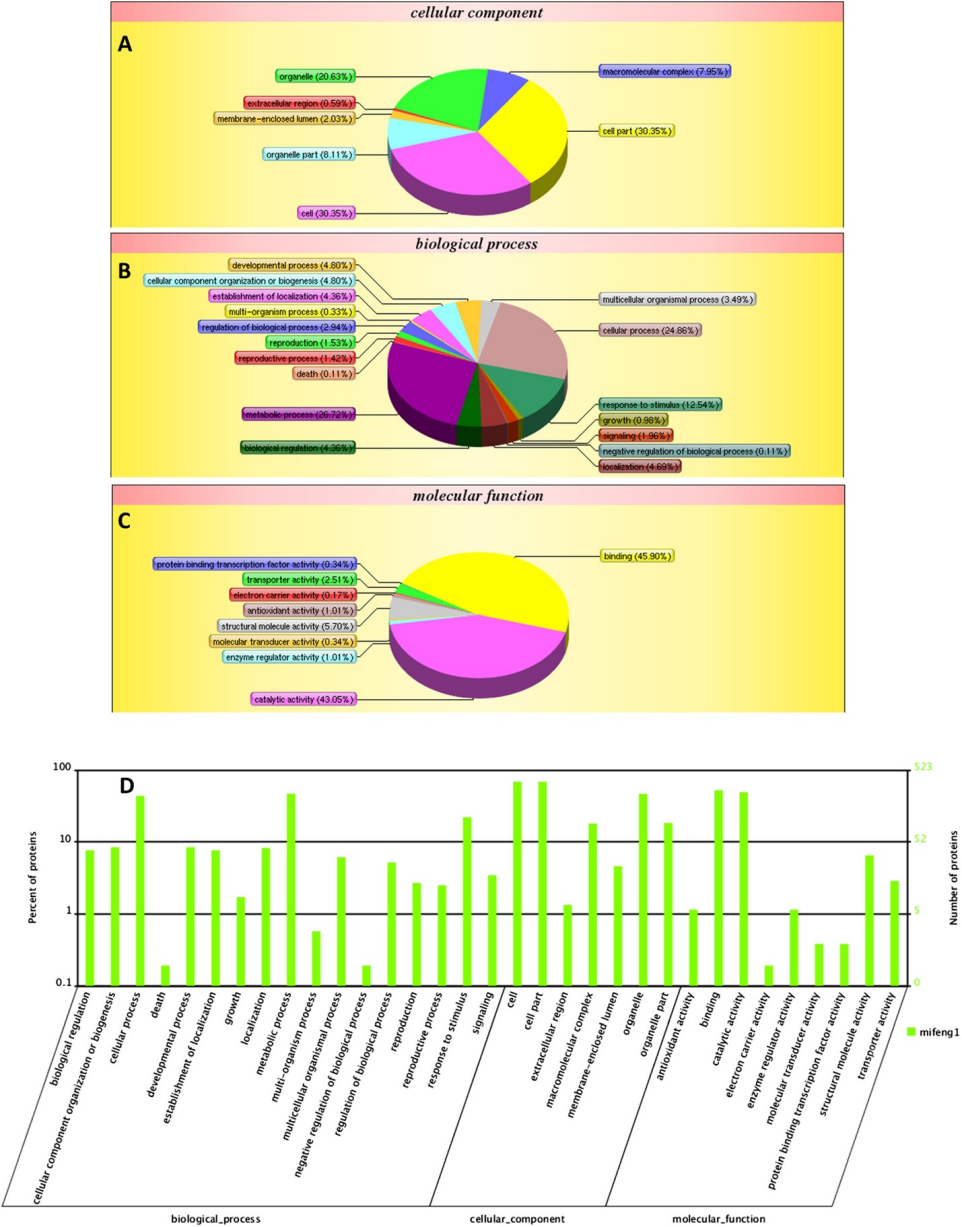
**Classification of GO categories.** The three functional categories were **(A)** cellular component, **(B)** biological process, and **(C)** molecular function. (D) WEGO output of our data. Hierarchical GO tree in which all the GO terms contained in the plot are shown to compare the annotation results. *x*-axis indicates functional items. *y*-axis (left) shows the percent of the proteins. *y*-axis (right) represents the number of proteins.

The final, selected DEPs were first analyzed using the GO database to determine their cellular component associations, molecular functions, and participation in biological processes. According to the functional properties, these proteins were classified into the following functional categories (Figure 2 (A-C)): binding (274, 45.90%), catalytic activity (257, 43.05%), cell & cell part (359, 30.35%), metabolic process (245, 26.72%), cellular process (228, 24.87%), organelle (244, 20.63%), response to stimulus (115, 12.54%), organelle part (96, 8.11%), macromolecular complex (94, 7.95%), structural molecule activity (34, 5.70%), and cellular component organization or biogenesis (44, 4.80%). Detailed information can be found in Additional file 3: File S4. Many of these functions play important roles in development and protein synthesis. Functional classification of the Clusters of Orthologous Groups (COGs) (Additional file 1: Figure S3, Additional file 3: File S5) was implemented to identify orthologous protein sets [49].

**Figure 3:**
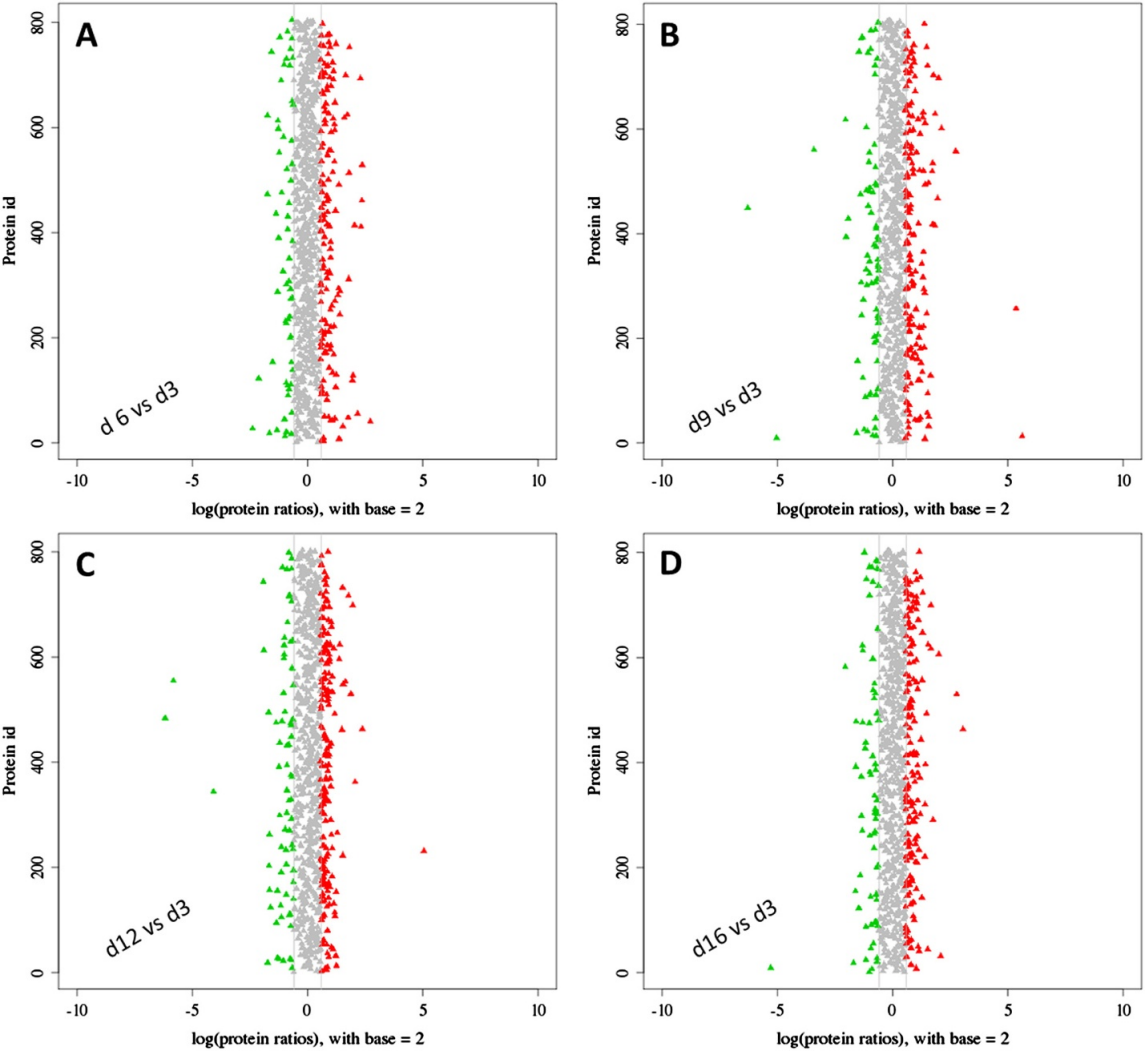
**Distribution of DEP abundances. (A-D)** DEPs at specific time points were relative to day 3 as the baseline. The *x*-axis indicates the fold-change (ratios) of proteins based on the logarithm with base 2. The *y*-axis indicates the protein ID. The candidate DEPs were indicated in red (up-regulated) or green (down-regulated), with the absolute value of log_2_ (protein fold-change) >1.5. The range of log-fold changes in panel B and C were much wider than panel A and D, revealed more DEPs change their expression pattern in d9 and d12 compared with d3 and d16. That may show us the right direction to focus their functions in the regulation of HG activity.

The dynamic range of DEP abundances are shown in Figure 3. Potential biomarkers are included in the red and green plots and represent various expression profiles. Many more proteins were significantly differentially expressed in day9 and d12 than d6 and d16. Such as down-regulated vitellogenin, apidermin and up-regulated troponin C type II b, 60S ribosomal protein L24, 40S ribosomal protein S8 in d9, down-regulated MRJP7, uncharacterized protein LOC100866317, *N*-acetylneuraminate lyase-like, and up-regulated ferritin, 60S ribosomal protein L24, ribosomal protein L17 isoform B (Additional file 3: File S3).

**Figure 4:**
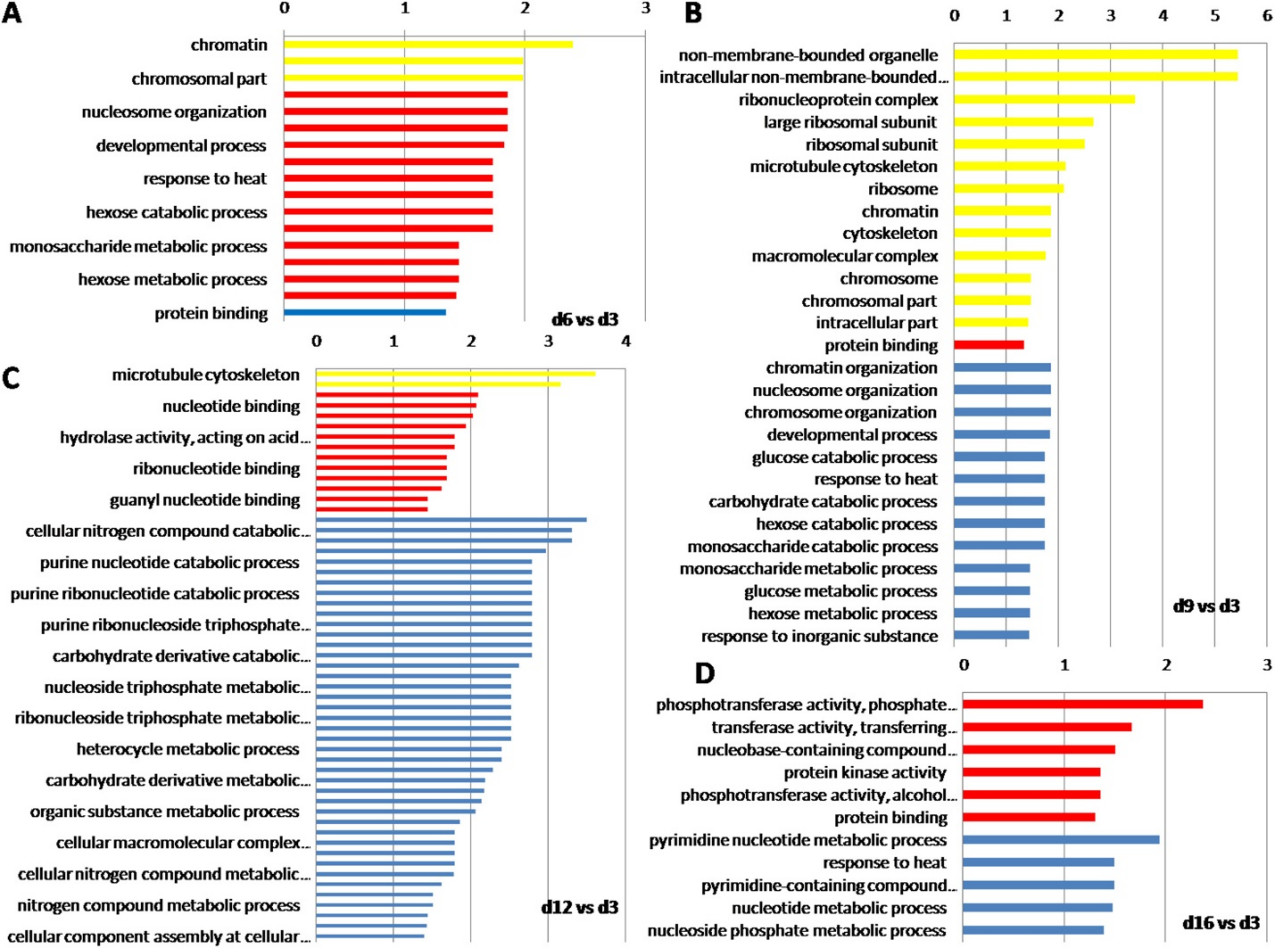
**GO enrichment analysis of DEPs.** Panel A to D represent the compared groups of day 6 vs day 3, day 9 vs. d3, day 12 vs. d3 and day 16 vs. d3. To the left of each plot: GO terms. Above each plot: -log_10_ (p-value). Yellow bars indicate cellular component, red bars represent a biological process, and blue bars denote a molecular function.

**Figure 5:**
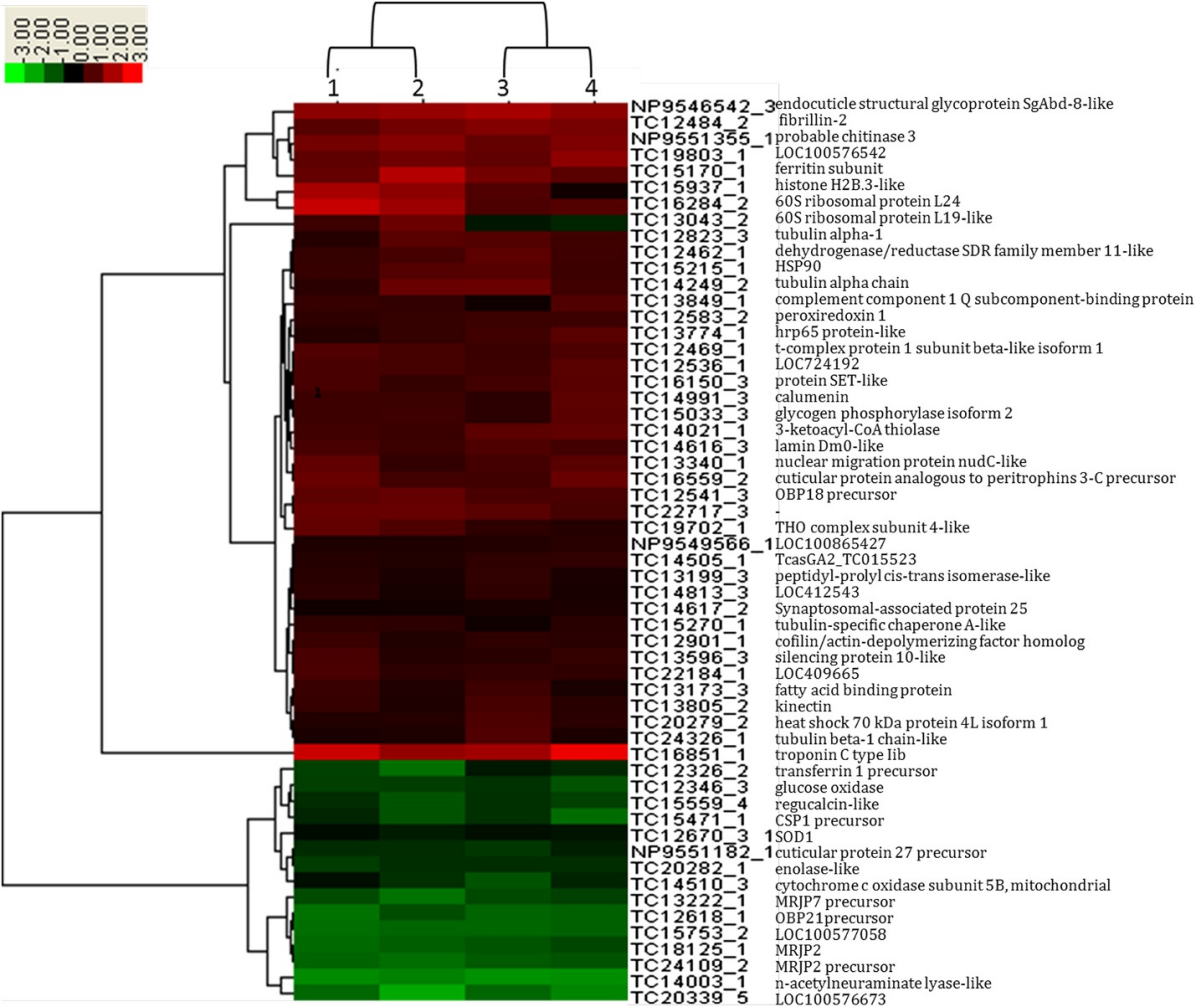
**Hierarchical Clustering of multiple samples (56 proteins in all).** Each column represents and compares a group relative to day 3, (1: d6 vs. d3; 2: d9 vs. d3; 3: d12 vs. d3; 4: d16 vs. d3) and each row represents a gene. Expression differences are shown in different colors; red indicates up-regulation, whereas green indicates down-regulation.

Furthermore, GO enrichment analysis (Figure 4, Additional file 3: File S4) and hierarchical clustering (Figure 5) and KEGG pathway analysis (Additional file 3: File S6) were illustrated to help us understand the results. Enriched functional terms shifted dramatically from d6 to d16, while there was rarely overlap in the enriched terms between the 5 time points indicating clear changes overtime in the regulation of HG activity. A hierarchical cluster analysis revealed two unambiguous clusters of proteins, characterized by up-regulation or down-regulation during the RJ secretion. Protein expressions in sample d9 have a wider range, compared with other samples (Figure 5, Additional file 3: File S3). Histone H2B-like (TC15937_1), a functional posttranslational modificator [50], elevated in the expression as primary requirement for cellular proliferation [51] and down-regulated exclusively in d16, indicating the cellular metabolism and replacement weaken in the later stage of RJ secretion. Coincidentally, ribosomal protein L19 (TC13043_2), strongly down-regulated in d12 and d16, demonstrated the decrease of protein synthesis rate in the cytoplasm over time.

To identify the biological pathways that were active in the HGs at the five time points, a total of 649 proteins were mapped to canonical signaling pathways found in KEGG. These proteins included 167 (25.73%) proteins that are involved in metabolic pathways (ko01100); 82 (12.63%) proteins that function in the biosynthesis of secondary metabolites (ko01110) and 74 (11.4%) proteins with roles in microbial metabolism in diverse environments (ko01120). The most enriched pathways are RNA transport (ko03013) in d6 vs. d3, ribosome (ko03010) in d9 vs. d3, gap junction (ko04540) in d12 vs. d3 and pathogenic Escherichia coli infection in (ko05130) d16 vs. d3, which are listed in Additional file 3: File S6.

### Relative qPCR analysis

**Figure 6:**
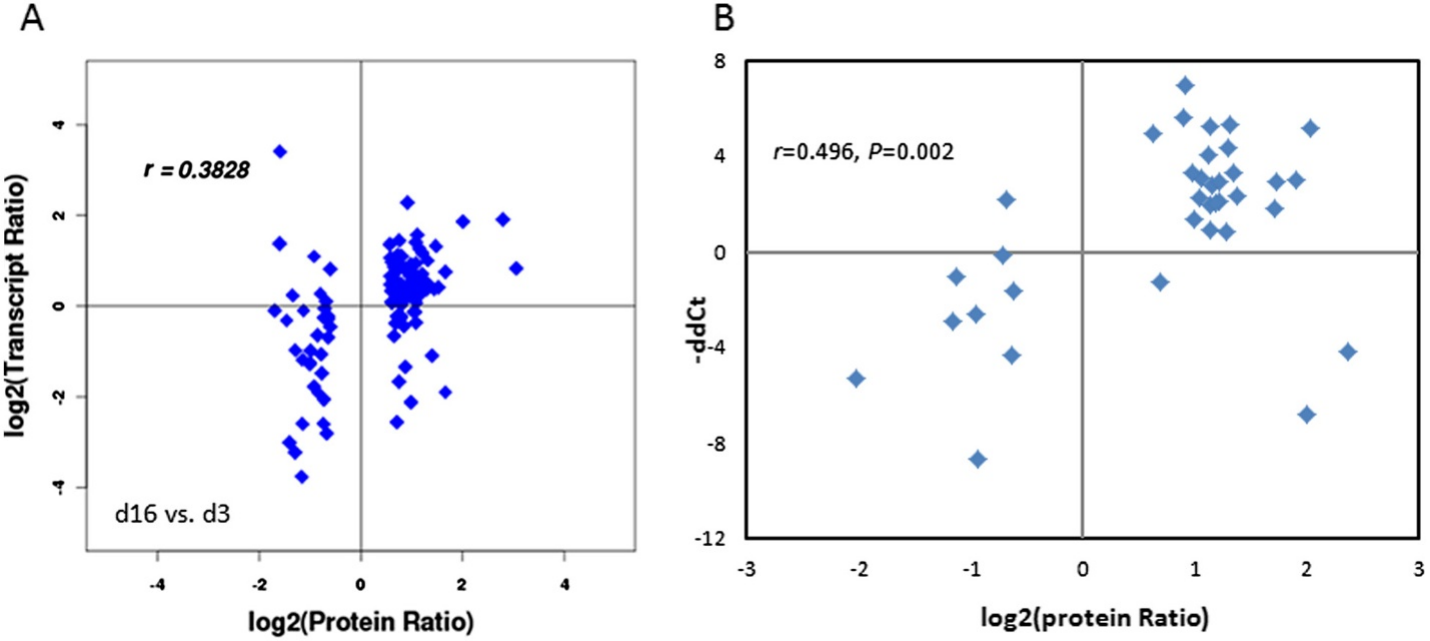
**Correlation between the expression levels of 35 proteins and their corresponding gene transcripts. (A)** The expression levels of proteins and their transcripts in d16 vs. d3 were used for a whole understanding to reveal the correlation between the two methodologies, showing the significant positive correlation (r = 0.3828, P = 0.010). **(B)** The qPCR results show the better correlation with r = 0.496, P = 0.002. The fold-change (ratios) of proteins and transcripts were based on the logarithm with base 2.

The expression values of genes corresponding to the DEPs were examined by qPCR (Figure 6). According to the published RNA-seq data (data not shown) of the same experimental design [41], the comparison between changes in transcript and protein expression in day16 vs. day3 revealed a significant positive correlation (*r* = 0.3828, *P* = 0.01).

## Discussion

A number of features of the honeybee have facilitated its genetic analysis, including easy rearing, a short life cycle, high fecundity, the public accessibility of genome information, and the simplicity of making genetic crosses [52]. The Honeybee Genome Project has provided a molecular basis for further research on the biological characteristics of honeybees, as well as the molecular mechanisms and evolution of their social behaviors. These features have allowed the honeybee to develop from an economic insect into a model organism that can be utilized for biological study [53]. RJ is a milky secretion that is produced by worker bees for use in the development and nurturing of queen and larvae.

We performed DEP profiling using iTRAQ followed by LC separation and MS/MS of the tagged, pooled peptides. For identification of metabolites, mass spectrometers that have a 3 ppm mass accuracy and a 2% error for isotopic abundance patterns outperform mass spectrometers that have less than 1 ppm mass accuracy or even a 0.1 ppm mass accuracy (calculated from simulations) but that do not include the isotopic patterns in the calculation of the molecular formulae [54].

Our results also show that the changes in protein expression throughout HG development can be related to different functions other than its traditional role in royal jelly production. The identified proteins can be divided into two categories: secreted and non-secreted proteins. The functions of the DEPs that were commonly expressed in the five samples (class **o**, Figure 1B and C) were selected for further discussion.

### DEPs associated with high HG secretion activity

HG secretion reaches its highest level between 6–12 days and decreases afterward [49, 50]. The polyfunctional properties of the secreted proteins may be demonstrated on proteins of larval diet, particularly proteins of RJ [55]. Secreted proteins up-regulated during this period include MRJPs (Additional file 3: File S2, Additional file 1: Figure S4), which account for most of the proteins in RJ. MRJPs are potentially involved in making queens. Up to 50% of RJ dry weight is composed of proteins and 82-90% of them belong to the major royal jelly protein family (MRJPs 1–9) [12, 14]. MRJP1 is the most abundant of them, representing 48% of the water-soluble proteins [15]. MRJPs are highly expressed in the HG, although they are also expressed in other tissues including the brain [56]. What is the molecular function of MRJPs? There are three lines of evidence that support that several members of the MRJP family evolved nutritionally related functions. First, MRJP3 contains repetitive pentapeptide regions abundant in nitrogen-rich amino acids that may function as deposits of biologically accessible nitrogen [56, 57]. Second, MRJP1 and MRJP2 composition is especially rich in essential amino acids [58]. Third, MRJP1 is critical during the nutritionally–mediated caste determination process [59]. MRJPs are likely involved in other nutritionally-related functions, besides their traditional role in larvae and queen nutrition. They are likely involved in the transfer of secreted proteins among workers by means of trophalaxis [60–62]. MRJP2 and MRJP7 are preferentially expressed in heads of sterile workers [63]. Overall, these results suggest that the MRJPs have an important role in the pleiotropic interactions between reproduction and nutrition in the regulation of worker’s division of labor. Surprisingly, the expression of most of the MRJPs in our study did not correlate with the period of high HG activity.

On the other hand, non-secreted proteins up-regulated during this period includes nanA (TC14003_1), which is involved in the utilization of *N*-acetylneuraminic acid [64]. Nitrogen is an essential and limiting component of biogenic polymers, such as nucleic acids and proteins, animals must obtain it from exogenous sources [56]. In the honeybee, it is possible that nanA contributes to amino acid metabolism. Fibrillin-1 (TC12484_2) is encoded by *fbn1* and plays an important role in building the connective tissue of the body [65]. We speculate that fibrillin-1 is important in the construction of the cytoskeleton. The dehydrogenase/reductase SDR family (*SDR*, TC12462_1) of proteins constitutes a large family of NADPH-dependent oxidoreductases that have critical roles in lipid, amino acid, carbohydrate, cofactor, hormone, and xenobiotic metabolism, as well as in redox sensor mechanisms [66]. It is now clear that SDRs represent one of the oldest protein families and contribute to essential functions and interactions in all domains of life, which highlights their versatility and fundamental importance in metabolic processes. Heat shock protein 90 (*HSP90*, TC15215_1) is an abundant and highly conserved molecular chaperone required for the stability and function of a number of conditionally activated and/or expressed signaling proteins, as well as multiple mutated, chimeric, or overexpressed signaling proteins; HSP90 may promote cell growth and/or survival [67, 68]. Currently identified HSPs in honeybees are induced either at high temperatures [68] or when the bee suffers from pathogenic infections [69]. Ferritin (TC15170_1) is associated with the vacuolar system and functions as an iron transporter in insects [70, 71]. It is found inside the rough endoplasmic reticulum in the iron-rich granules of the fat body of the honeybees [72].

Unexpectedly, we found some proteins apparently not related with HG function. This includes odorant-binding protein 21 precursor (TC12618_1) which has been involved in recognition of chemical stimuli in the olfactory system and is normally expressed in the antennae of foragers [73, 74]. A high level of expression in the HG suggests that this protein may have additional unknown functions.

### Proteins that are exclusively expressed at day 6, 9, 12, or 16 relative to day 3

There were 38 proteins (27 of which were up-regulated and 11 of which were down-regulated) that were preferentially expressed during the early stage of HG development (day 6, class **a** in Figure 1B and Additional file 2: Table S2). The matured morphological structures are conducive to protein synthesis and secretion [75, 76]. Thus, 40S ribosomal protein S8 (TC12671_2) was significantly increased relative to other proteins. Similarly, chaperonin subunit 6a zeta (TC14834_3) might help in the folding of macromolecular structures and the assembly or disassembly of proteins.

The 35 proteins (23 of which were up-regulated and 12 of which were down-regulated) that were preferentially expressed at the peak of HG activity (day 9, class **b** in Figure 1B) likely play a role in protein synthesis and RJ secretion. The expression of ribosomal protein L23A (TC12633_1) and ribosomal protein L18e (TC19595_1) were significantly increased relative to other proteins. These proteins may therefore play a critical role in the process of RJ initiation and secretion.

There were 57 proteins (34 of which were up-regulated and 23 of which were down-regulated) that were preferentially expressed on day 12 (class **c** in Figure 1B). The shrinkage of HGs at this stage may bring about a decline in RJ production.

There were 52 proteins (35 of which were up-regulated expressed and 17 of which were down-regulated) that were preferentially expressed on day 16 (class **d** in Figure 1B). At this late stage, the worker bees stop their production of RJ and become forager bees to execute other important tasks. Proteins preferentially expressed in this stage include α-glucosidase, glucose oxidase, and alpha α-amylase, which are members of the same family of enzymes and catalyze the hydrolysis of the glucosidic linkages of starch [77].

### Gene ontology annotations of DEPs in abundance

The overview of the subcellular location and biological processes of categorization of these proteins was performed on the basis of Gene Ontology (GO) annotations with the percentage contribution of each category (Figure 2) [78]. As shown in Figure 4, cellular component and molecular function ontology revealed that the majority of the identified DEPs were enriched in the samples d9 and d12 (Figure 4B and C), compared to the molecular function ontology barely enriched in samples d6 and d16 (Figure 4A and D). The results indicated that the HG activity was associated with multiple genes regulation, in which the molecular function related proteins might play the important roles.

## Conclusions

Overall, the changes in abundance of DEPs that were observed in this study may indicate that there are major proteins that can monitor and regulate the initiation, development, and degeneration of HGs by modulating and coordinating the amounts of the corresponding proteins. The characterization of dynamic protein networks through the use of proteomics analyses could help us understand the molecular events that occur during HG development. Thus, we produced a comprehensive view of how HGs differentially and developmentally express genes and secrete proteins and other molecules according to age and stage of development.

## Electronic supplementary material

Additional file 1: Figure S1-S4: **Figure S1**, protein concentration and integrity. **Figure S2**, the identified spectra and proteins statistics. **Figure S3**, COG functional classification of the samples. **Figure S4**, MRJPs family expression levels. (ZIP 2 MB)

Additional file 2: Table S1-S3: **Table S1**, Statistics of samples. **Table S2**, Genes specifically expressed in the four groups. **Table S3**, Expression profiling of DEPs common among the samples. (DOCX 43 KB)

Additional file 3: File S1-S6: **File S1**, Gene-specific primers and expression levels. **File S2**, overall_annot proteins quantified from iTRAQ experiment. **File S3**, up_down of DEPs. **File S4**, GO2protein. **File S5**, cog2protein. **File S6**, pathway enrichment. (ZIP 2 MB)

## Abbreviations

HG: Hypopharyngeal gland
iTRAQ: Isobaric tag for relative and absolute quantification
DEP: Differentially expressed protein
MS: Mass spectrometry
TOF: Time of flight
DIGE: Difference gel electrophoresis
IAM: Iodoacetamide
TEAB: Tetraethyl-ammonium bicarbonate
DTT: Dithiothreitol
Ppm: Parts per million.
