# Supplementary material for: Proteomics analysis reveals protein expression differences for hypopharyngeal gland activity in the honeybee, Apis mellifera carnica Pollmann
Source: BMC Genomics. 2014 Aug 8;15(1):665. doi: 10.1186/1471-2164-15-665 (PMC4141115; doi:10.1186/1471-2164-15-665)
Supplement: Supplementary file 2 — Additional file 2: Table S1-S3: Table S1, Statistics of samples. Table S2, Genes specifically expressed in the four groups. Table S3, Expression profiling of DEPs common among the samples. (DOCX 43 KB) [file 12864_2014_6363_MOESM2_ESM.docx]

**Additional file 2: Table S1 Statistics of samples**

| Sample ID | 1 | 2 | 3 | 4 | 5 |
| --- | --- | --- | --- | --- | --- |
| Age (Day) | 3 | 6 | 9 | 12 | 16 |
| Total protein (μg) | 881.4 | 1093.8 | 1047.6 | 913.2 | 859.5 |
| Total Spectra | 193,671* |  |  |  |  |
| Spectra | 9,660 |  |  |  |  |
| Unique Spectra | 9,177 |  |  |  |  |
| Peptide | 3,880 |  |  |  |  |
| Unique Peptide | 3,757 |  |  |  |  |
| Protein | 1,282 |  |  |  |  |

*Results of raw data searched against the database created from the tanscriptomic CDS FASTA database (GEO accession number: GSE47136) by six-frame translation (34702 sequences) with Mascot software (Matrix Science, London, U.K.; version 2.3.02).

**Additional file 2: Table S2 Genes specifically expressed in the four groups.**

| Gene ID | Full name |
| --- | --- |
| **a** | **d6 vs. d3** |
| TC15281_2 | Zinc finger protein |
| TC14649_1 | Malate dehydrogenase |
| TC20282_1 | Enolase |
| TC13168_2 | CG33950-PG, isoform G |
| TC15820_1 | UV excision repair protein RAD23 homolog B |
| TC12322_2 | Prophenoloxidase |
| TC22456_2 | Melittin precursor |
| TC19674_2 | AGAP007975-PA |
| TC13706_3 | Venom protein 8 |
| TC15726_3 | LRR protein WM1.10 |
| TC14781_2 | Fructose-bisphosphate aldolase |
| TC13538_3 | GA18354-PA |
| NP9543885_1 | GB\|XM_001122412.1\|XP_001122412.1 similar to prefoldin subunit 1 |
| NP9550883_1 | GB\|XM_392361.3\|XP_392361.3 similar to CG33519-PB isoform 1 |
| NP9548063_1 | GB\|XM_397496.3\|XP_397496.2 hypothetical protein |
| BI505191_2 | Kinesin light chain 1 |
| NP9544933_1 | GB\|XM_001121022.1\|XP_001121022.1 similar to CG32446-PA |
| TC14687_2 | _ |
| TC14626_1 | AGAP004492-PA |
| TC16621_2 | _ |
| TC16504_1 | _ |
| NP9549336_1 | GB\|XM_001122667.1\|XP_001122667.1 similar to CG13214-PA, isoform A |
| TC16895_1 | Acyl carrier protein |
| TC21290_3 | Synapsin isoform 2.1 |
| TC12901_1 | AGAP012056-PA |
| TC13916_1 | Odorant binding protein ASP2 |
| TC19665_1 | _ |
| NP9553895_1 | GB\|XM_396457.1\|XP_396457.1 similar to CG8578-PA |
| TC15002_2 | _ |
| NP9546861_1 | GB\|XM_001122907.1\|XP_001122907.1 similar to Ccp84Ad CG2341-PA |
| TC13352_1 | Histone H2A |
| TC14834_3 | Chaperonin subunit 6a zeta |
| TC13085_1 | AGAP001754-PA |
| NP9553268_1 | GB\|XM_624678.1\|XP_624681.1 similar to cabeza CG3606-PB, isoform B |
| TC22103_2 | Isoform A of Q27294 |
| TC12671_2 | 40S ribosomal protein S8 |
| TC13195_1 | Mitochondrial ribosomal protein L12 |
| TC16805_3 | Heterogeneous nuclear ribonucleoprotein A1, A2/B1 homolog |
|  | ` |
| **b** | **d9 vs. d3** |
| TC20864_2 | CG5174-PA, isoform A |
| TC14745_3 | AGAP000745-PA |
| TC16998_1 | AGAP001364-PA |
| BP539811_6 | Major royal jelly protein 4 precursor |
| TC14608_2 | Isochorismatase family protein |
| TC21572_1 | Chymotrypsin inhibitor |
| TC13522_2 | cation diffusion facilitator family transporter containing protein |
| TC16862_1 | sAGAP010539-PA |
| TC12375_2 | Major royal jelly protein 4 precursor |
| TC12665_1 | Venom carbohydrate-rich protein precursor |
| TC22535_1 | ATPase inhibitor-like protein |
| TC23147_3 | Defensin 1 |
| TC22854_3 | CG10851-PC, isoform C |
| NP9548589_1 | GB\|XM_391860.3\|XP_391860.3 similar to B52 CG10851-PB, isoform B |
| TC23992_2 | AGAP004592-PA; n=1 |
| TC12633_1 | Ribosomal protein L23A |
| TC15095_6 | _ |
| TC13791_1 | AGAP003584-PA |
| TC13313_1 | Eukaryotic translation initiation factor |
| TC13459_2 | Ribosomal protein L9e |
| TC17835_1 | Malic enzyme |
| TC16021_3 | Ribosomal protein L22e |
| DB731285_1 | Ribosomal protein L22 |
| TC13168_1 | CG33950-PG, isoform G |
| TC17156_1 | Ribosomal protein S20 |
| TC21473_2 | LOC100036668 protein |
| TC14454_2 | CG9090 |
| TC12930_3 | AGAP005076-PB |
| TC19595_1 | Ribosomal protein L18e |
| TC15149_2 | Protein kinase C substrate 80K-H |
| TC14010_2 | Histone H2A |
| TC17292_1 | Histone H2A |
| NP9546647_1 | GB\|XM_001119899.1\|XP_001119899.1 similar to CG31618-PA |
| NP9552465_1 | GB\|XM_001120346.1\|XP_001120346.1 similar to CG31618-PA |
| NP9552500_1 | GB\|XM_001120934.1\|XP_001120934.1 similar to CG31618-PA |
|  | ` |
| **c** | **d12 vs. d3** |
| NP9552724_1 | GB\|XM_394212.3\|XP_394212.3 similar to F36G9.12 |
| TC17627_2 | E3 component of acetoin dehydrogenase enzyme system |
| TC20567_3 | Cytochrome c oxidase subunit VIb |
| NP9553116_1 | GB\|XM_624144.1\|XP_624147.1 similar to CG3560-PA |
| TC14733_3 | AGAP007841-PA |
| TC14837_1 | Cytochrome c oxidase subunit Va |
| NP9551182_1 | GB\|XM_001121346.1\|XP_001121346.1 similar to CG15884-PA |
| TC16817_3 | Cuticular protein 125, RR-1 family |
| TC16485_3 | GH01093p |
| TC23213_4 | Tropomyosin |
| TC16172_1 | _ |
| TC23256_3 | Troponin I isoform 6b1 |
| TC17099_1 | _ |
| TC22950_3 | Carbonic anhydrase |
| TC24042_3 | Troponin I isoform 6b2 |
| TC19792_1 | Glycerol-3-phosphate dehydrogenase |
| TC15188_3 | Myosin light chain |
| TC14683_3 | Chromosome chr13 scaffold_286, whole genome shotgun sequence |
| TC15274_2 | _ |
| TC14510_3 | AGAP008724-PA |
| TC18427_2 | troponin C type IIIa |
| TC12355_3 | Major royal jelly protein 9 |
| NP9544686_2 | GB\|XM_001123307.1\|XP_001123307.1 similar to NADH-ubiquinone oxidoreductase 18 kDa subunit, mitochondrial precursor (Complex I-18 kDa) (CI-18 kDa) (Complex I-AQDQ) (CI-AQDQ) |
| TC13199_3 | Peptidyl-prolyl cis-trans isomerase |
| TC15546_1 | AGAP004335-PA |
| TC16445_1 | Beta chain spectrin |
| NP9548294_1 | GB\|XM_394961.3\|XP_394961.3 similar to CG9485-PA, isoform A |
| TC14618_2 | Isocitrate dehydrogenase |
| NP9550918_1 | GB\|XM_396472.3\|XP_396472.3 similar to Papilin CG33103-PB, isoform B isoform 1 |
| TC20279_2 | AGAP010331-PA |
| TC15615_1 | AGAP003296-PA |
| TC15465_3 | Dynactin subunit 2 |
| TC16187_2 | AGAP005963-PA |
| TC15703_2 | Dihydropyrimidinase |
| TC22609_2 | AGAP011396-PA |
| TC15502_1 | Glutamate carboxypeptidase |
| NP9548984_1 | GB\|XM_393694.3\|XP_393694.3 similar to Limpet CG32171-PD, isoform D |
| TC24100_2 | _ |
| TC13082_1 | CG17838-PH, isoform H |
| NP9553875_1 | GB\|XM_397380.1\|XP_397380.1 similar to Ef1-like factor CG6382-PA |
| TC20043_2 | Thymosin beta |
| TC14078_1 | ATP-citrate synthase |
| TC20522_1 | ATP-citrate synthase |
| TC12491_1 | AGAP007474-PA |
| TC13012_1 | homologue to Heat shock cognate 70 |
| TC13926_3 | homologue to AGAP007942-PA |
| TC18431_3 | Sjogren syndrome antigen B |
| TC15595_3 | _ |
| TC12973_1 | Predicted protein |
| TC13973_3 | AGAP008604-PA |
| TC15535_2 | homologue to 26S protease regulatory subunit |
| NP9549605_1 | GB\|XM_397120.3\|XP_397120.3 similar to CG11142-PA, isoform A isoform 1 |
| TC12717_2 | Protein transport protein sec23 |
| TC20162_2 | Elongation factor 1-alpha |
| TC24326_1 | Beta-tubulin |
| TC13770_3 | Nidogen |
| TC14088_1 | AGAP007349-PA |
|  |  |
| **d** | **d16 vs. d3** |
| TC18296_3 | Cytidylate kinase |
| TC13954_1 | _ |
| TC21873_2 | _ |
| TC14700_3 | _ |
| TC13838_3 | CG32130-PE, isoform E |
| TC18404_2 | AGAP005807-PA |
| TC13378_2 | AGAP008165-PA |
| TC20481_3 | AGAP008165-PA |
| TC15753_1 | IP16036p |
| TC20330_1 | _ |
| TC20200_3 | Flagelliform silk protein-1 |
| TC12359_3 | Alpha-glucosidase |
| TC20807_2 | Alpha-glucosidase precursor |
| TC17144_3 | GA16231-PA |
| TC15057_3 | Phosphatidylinositol-binding clathrin assembly protein |
| TC14567_3 | GA17497-PA |
| TC14003_2 | _ |
| TC13433_1 | homologue to Arginine/serine-rich splicing factor |
| TC13329_3 | _ |
| TC13054_3 | Nucleoside diphosphate kinase |
| TC15747_2 | Myelinprotein expression factor |
| NP9543801_2 | GB\|XM_001120518.1\|XP_001120518.1 similar to CG30045-PA |
| TC13163_3 | Hsc70/Hsp90-organizing protein HOP |
| TC13327_2 | _ |
| NP9552331_1 | GB\|XM_394154.3\|XP_394154.3 similar to dystrophin CG31175-PA, isoform A |
| TC23177_2 | Apolipophorins precursor [Contains: Apolipophorin-2 (Apolipophorin II) (apoLp-2); Apolipophorin-1 (Apolipophorin I) (apoLp-1)] |
| TC13656_3 | Translation elongation factor-1 gamma |
| TC14095_2 | Rho GDP-dissociation inhibitor |
| BI504770_2 | Rho GDP-dissociation inhibitor |
| TC14505_1 | homologue to AGAP005507-PA |
| TC18598_2 | homologue to AGAP005507-PA |
| TC16548_1 | homologue to AGAP005507-PA |
| TC23752_2 | AGAP005507-PA |
| DB730347_2 | homologue to AGAP005507-PA |
| TC15481_2 | _ |
| BI504734_3 | Sex-lethal |
| TC19476_1 | Predicted membrane protein |
| TC15834_1 | AGAP003037-PA |
| TC13189_3 | Transgelin; n=1; Bombyx mori\|Rep: Transgelin - Bombyx mori (Silk moth), complete |
| TC12486_3 | _ |
| TC19597_2 | AGAP010419-PA |
| TC12481_3 | homologue to Myosin regulatory light chain 2 smooth muscle |
| TC12852_3 | _ |
| TC15621_1 | Myosin 2 light chain |
| TC21743_1 | AGAP007620-PA |
| TC12777_1 | GA16365-PA |
| TC16168_2 | AGAP012235-PA |
| TC21339_3 | _ |
| TC14808_2 | Transcription elongation factor S-II |
| TC15426_3 | Peptidyl-prolyl cis-trans isomerase |
| TC16240_2 | Peptidyl-prolyl cis-trans isomerase |
| DB729296_2 | Peptidyl-prolyl cis-trans isomerase |
|  |  |
| **e** | **d6 vs. d3 and d9 vs. d3** |
| TC12326_2 | Transferrin |
| BI502735_1 | Pyruvate dehydrogenase |
| NP9549010_1 | GB\|XM_001121882.1\|XP_001121882.1 similar to ATPase coupling factor 6 CG4412-PA |
| TC15789_3 | Ribosomal protein L8 |
| TC19702_1 | RNA and export factor binding protein; |
| NP9550868_1 | GB\|XM_001122212.1\|XP_001122212.1 similar to mitochondrial ribosomal protein L54 CG9353-PA |
| NP9552473_1 | GB\|XM_001121111.1\|XP_001121111.1 similar to Histone H1 |
| TC14067_1 | Phospholipid-hydroperoxide glutathione peroxidase |
| TC13043_2 | Ribosomal protein L19 |
| TC13004_1 | S-phase kinase-associated protein |
| TC19365_3 | Predicted protein |
|  |  |
| **f** | **d6 vs. d3 and d12 vs. d3** |
| TC20818_1 | Dopamine D4 |
| TC20616_1 | OBP3 |
| TC13805_2 | _ |
| TC13601_1 | _ |
| TC16380_1 | CG33715-PB, isoform B |
| NP9546135_1 | GB\|XM_001122457.1\|XP_001122457.1 similar to Laminin B1 CG7123-PA, isoform A |
| TC13173_3 | Fatty acid binding protein |
| TC20289_2 | Muscle LIM protein |
| TC20445_3 | Troponin I isoform 6a1 |
| TC24102_3 | Troponin I isoform 6a1 |
| TC22184_1 | CG33521-PC, isoform C |
| TC13444_1 | _ |
|  |  |
| **g** | **d6 vs. d3 and d16 vs. d3** |
| TC16464_3 | apidermin 2 |
| TC15304_1 | _ |
| BI506587_3 | _ |
| TC18352_3 | CG11889-PA, isoform A |
| TC19239_3 | GA21834-PA |
| TC12736_1 | ATP-dependent RNA helicase |
| TC14239_1 | _ |
| TC15029_2 | Protein FAM10A4 |
| NP9550624_3 | GB\|XM_001121945.1\|XP_001121945.1 similar to CG17052-PA |
| TC13596_3 | Predicted protein |
| NP9547232_1 | GB\|XM_001122227.1\|XP_001122227.1 similar to CG6180-PA |
|  |  |
| **h** | **d9 vs. d3 and d12 vs. d3** |
| TC12657_3 | Ymf71 |
| NP9553889_1 | GB\|XM_396653.1\|XP_396653.1 similar to Tropomyosin-2 (Tropomyosin I) |
| TC18817_1 | Tubulin beta chain |
| TC13817_3 | CG18069-PE, isoform E |
| TC13847_2 | Endoplasmin |
| TC15207_2 | AGAP000561-PA |
| TC12593_2 | Spermatogenesis associated factor |
| TC18074_3 | DnaJ homolog subfamily A member 1 |
| TC12894_2 | AGAP003021-PA |
|  |  |
| **i** | **d9 vs. d3 and d16 vs. d3** |
| TC15980_1 | CG30084-PF, isoform F |
| TC14338_2 | Ribosomal protein S28e-like protein |
| TC15034_2 | _ |
| DB728232_2 | Major royal jelly protein 7 |
| TC15471_1 | chemosensory protein 1 |
| TC12344_3 | Major royal jelly protein 5 precursor |
| TC15559_4 | Uncharacterized protein Saci_1674 |
| DB730187_1 | _ |
| TC15267_2 | Glycosyl-phosphatidyl-inositol-anchored protein |
| TC17698_1 | AGAP005162-PB |
| TC13355_2 | Actin |
| TC17160_2 | Actin |
| TC23177_1 | Apolipophorins precursor [Contains: Apolipophorin-2 (Apolipophorin II) (apoLp-2); Apolipophorin-1 (Apolipophorin I) (apoLp-1)] |
| TC12741_2 | Protein disulfide isomerase |
| TC16430_2 | GA19141-PA |
| TC14457_3 | Ribosomal protein L4 |
| TC16175_3 | Ribosomal protein L17 isoform B |
| NP9545951_1 | GB\|XM_001120602.1\|XP_001120602.1 similar to Muscle protein 20 CG4696-PA, isoform A |
| TC14757_3 | 3-hydroxyacyl-CoA dehydrogenase |
| TC12908_1 | Ribosomal protein S2 |
|  |  |
| **j** | **d12 vs. d3 and d16 vs. d3** |
| TC21817_1 | Troponin I isoform 6a2 |
| TC13712_3 | _ |
| TC19890_1 | CG3777-PA, isoform A |
| TC13691_1 | Eukaryotic translation initiation factor 4A |
| TC14850_2 | AGAP011050-PA |
| TC15763_3 | T-complex protein 1, alpha subunit |
| TC13727_2 | Prohibitin |
| BP873771_2 | Prohibitin |
|  |  |
| **k** | **except d16 vs. d3** |
| TC16789_1 | Cobalamin biosynthesis protein CbiD |
| TC22478_1 | PaxB |
| TC17580_1 | _ |
| TC22404_2 | Major royal jelly protein MRJP1 precursor |
| TC15937_1 | _ |
| NP9549991_1 | GB\|XM_001120014.1\|XP_001120014.1 similar to Histone H2B |
| TC12893_1 | _ |
| NP9546648_1 | GB\|XM_001119846.1\|XP_001119846.1 similar to Histone H2B |
| TC16736_2 | GA18075-PA |
| TC13884_1 | AGAP005662-PA |
|  |  |
| **l** | **except d12 vs. d3** |
| TC12346_3 | Glucose oxidase |
| TC15033_3 | _ |
| TC24375_3 | _ |
| TC20432_3 | T-complex protein 1, delta subunit |
| NP9552348_1 | GB\|XM_001122335.1\|XP_001122335.1 similar to nucleolin |
| TC18264_1 | Ribosomal protein L14 |
| TC22105_3 | Ribosomal protein L14 |
| TC18871_1 | AGAP004993-PA |
| TC12776_3 | 60S ribosomal protein L13 |
| BI510391_3 | 60S ribosomal protein L13 |
| TC13849_1 | Complement component 1 q subcomponent binding protein-like protein |
| TC14991_3 | Cardiac calumenin |
| TC21275_5 | HSBP1-like protein |
| TC23479_2 | HSBP1-like protein |
|  |  |
| **m** | **except d9 vs. d3** |
| BI510835_2 | IP16036p |
| TC12290_3 | Vitellogenin precursor |
| TC16805_2 | Heterogeneous nuclear ribonucleoprotein A1, A2/B1 homolog |
| TC14272_1 | Myosin heavy chain, nonmuscle or smooth muscle |
|  |  |
| **n** | **except d6 vs. d3** |
| NP9546716_1 | GB\|XM_001123191.1\|XP_001123191.1 similar to stunted CG9032-PA, isoform A |
| TC12382_2 | Tetraspanin F139 |
| TC13254_3 | Ribosomal protein S3 |
| TC12823_3 | Alpha-tubulin |
| TC15616_1 | Alpha-tubulin |
| TC13774_1 | Splicing factor proline-and glutamine-rich |
| TC13152_2 | Vesicle amine transport protein |
| TC22154_1 | OBP13 |
| TC14249_2 | Tubulin alpha chain |
| TC12583_2 | Thiol peroxiredoxin |
| TC12710_2 | Heat-shock protein 70 |
|  |  |
| **o** | **in common** |
| TC24109_2 | Major royal jelly protein 2 precursor |
| TC15753_2 | IP16036p |
| TC13222_1 | Major royal jelly protein 7 |
| TC12618_1 | OBP21 |
| TC20339_5 | _ |
| TC18125_1 | Major royal jelly protein 2 precursor |
| TC24109_3 | Major royal jelly protein 2 precursor |
| TC14003_1 | _ |
| TC13340_1 | _ |
| TC12541_3 | OBP18 |
| TC20849_1 | OBP18 |
| TC16284_2 | 60S ribosomal protein L24 |
| TC12536_1 | _ |
| TC16150_3 | SET translocation) |
| TC12484_2 | _ |
| TC19803_1 | _ |
| TC16851_1 | troponin C type IIb |
| TC14616_3 | AGAP011938-PA |
| TC14021_1 | AGAP006821-PA |
| TC16559_2 | Obstractor C1 |
| TC12469_1 | AGAP009694-PA |
| TC12462_1 | _ |
| TC22717_3 | Per-hexamer repeat protein 5 |
| TC15215_1 | Heat shock protein 90 |
| NP9551355_1 | GB\|XM_001120217.1\|XP_001120217.1 similar to CG17052-PA |
| TC15170_1 | Ferritin |
| TC15364_3 | Ferritin |
| NP9546542_3 | GB\|XM_001122709.1\|XP_001122709.1 similar to CG8515-PA |

**Additional file 2: Table S3 Expression profiling of DEPs common among the samples (25 elments in all)**

| # | Accession | description | Ratio(d6/d3) | Ratio(d9/d3) | Ratio(d12/d3) | Ratio(d16/d3) |
| --- | --- | --- | --- | --- | --- | --- |
| 1 | TC24109_2 | MRJP2 | 2.2675737 | 2.320186 | 2.09205 | 1.988072 |
| 2 | TC15753_2 | PDZ and LIM domain protein 3 | 2.47524752 | 2.320186 | 2.320186 | 2.207506 |
| 3 | TC13222_1 | MRJP7 | 1.85873606 | 2.544529 | 1.845018 | 1.692047 |
| 4 | TC12618_1 | OBP21 | 2.55102041 | 1.883239 | 2.277904 | 2.212389 |
| 5 | TC20339_5 | LOC100866317 | 2.35294118 | 3.984064 | 2.331002 | 2.985075 |
| 6 | TC18125_1 | MRJP2 | 2.37529691 | 2.173913 | 2.004008 | 1.785714 |
| 7 | TC14003_1 | n-acetylneuraminate lyase-like | 3.10559006 | 2.906977 | 3.278689 | 3.205128 |
| 8 | TC13340_1 | nuclear migration protein nudC-like | 0.45187528 | 0.661813 | 0.568505 | 0.481232 |
| 9 | TC12541_3 | OBP18 | 0.46339203 | 0.438982 | 0.542594 | 0.542594 |
| 10 | TC16284_2 | 60S ribosomal protein L24 | 0.19758941 | 0.276396 | 0.535332 | 0.49776 |
| 11 | TC12536_1 | LOC724192 | 0.54824561 | 0.567859 | 0.604961 | 0.482393 |
| 12 | TC16150_3 | SET translocation | 0.54914882 | 0.652316 | 0.574713 | 0.483559 |
| 13 | TC12484_2 | fibrillin-1 | 0.48875855 | 0.390472 | 0.342583 | 0.374532 |
| 14 | TC19803_1 | LOC552071 | 0.45787546 | 0.398406 | 0.450248 | 0.312598 |
| 15 | TC16851_1 | troponin C type IIb | 0.19124116 | 0.296736 | 0.268168 | 0.14374 |
| 16 | TC14616_3 | lamin Dm0-like | 0.54914882 | 0.613497 | 0.524934 | 0.576369 |
| 17 | TC14021_1 | 3-ketoacyl-CoA thiolase, mitochondrial-like | 0.58513751 | 0.621891 | 0.470146 | 0.449438 |
| 18 | TC16559_2 | cuticular protein analogous to peritrophins 3-C precursor | 0.42625746 | 0.581395 | 0.582751 | 0.42123 |
| 19 | TC12469_1 | t-complex protein 1 subunit beta-like isoform 1 | 0.51177073 | 0.564972 | 0.619195 | 0.52521 |
| 20 | TC12462_1 | dehydrogenase/reductase SDR family member 11-like | 0.64599483 | 0.563698 | 0.467727 | 0.600962 |
| 21 | TC22717_3 | Per-hexamer repeat protein 5 | 0.43327556 | 0.428449 | 0.479846 | 0.566893 |
| 22 | TC15215_1 | Heat shock protein 90 | 0.63613232 | 0.500501 | 0.487567 | 0.605327 |
| 23 | NP9551355_1 | LOC724382 | 0.38491147 | 0.335345 | 0.436872 | 0.355366 |
| 24 | TC15170_1 | Ferritin | 0.43668122 | 0.226912 | 0.381098 | 0.481928 |
| 25 | NP9546542_3 | endocuticle structural glycoprotein SgAbd-8-like | 0.31416902 | 0.289184 | 0.254194 | 0.312989 |
